# Enhancing Rangatahi Wellbeing in Secondary Education Through Implementation of the Meke Meter™

**DOI:** 10.1007/s40841-025-00390-6

**Published:** 2025-06-02

**Authors:** Dana Armstrong, Anita Jagroop-Dearing, Rachel H. Forrest

**Affiliations:** 1 Faculty of Education, Humanities and Health Science, Eastern Institute of Technology, Hawke’s Bay, New Zealand; 2 The Meke Foundation, Hawke’s Bay, New Zealand; 3 College of Health, Massey University, Wellington, New Zealand

**Keywords:** Adolescent, Aotearoa New Zealand, Rangatahi, Secondary education, Student, Wellbeing

## Abstract

Adolescent wellbeing is crucial, yet secondary schools face challenges in effectively addressing it. The Meke Meter™, an indigenous self-reflection tool, shows promise in promoting wellbeing, but its application in secondary schools remains unexplored. This study investigates the suitability and efficacy of the Meke Meter™ in capturing the self-evaluated wellbeing of rangatahi (adolescents) in Aotearoa New Zealand secondary schools. A qualitative multiple case study design was employed, involving two case studies: one in a mainstream co-educational school using the paper-based Meke Meter™, and another in an alternative education program using the online version. Data collection involved questionnaires for students and teachers, analysed through inductive thematic analysis and appreciative inquiry. Both students and teachers found the Meke Meter™ appealing and valuable for self-reflection and goal-setting. The tool’s ease of use, visual design, and alignment with the curriculum were highlighted. It facilitated pastoral care, student voice, and co-construction of learning. However, challenges in data tracking and the need for clearer explanations and additional resources were identified. The Meke Meter™ shows promise in promoting rangatahi wellbeing in secondary schools. Future development should focus on a mobile app with goal-setting, gamification, and notification features. Comprehensive consultation with stakeholders is crucial to ensure cultural safety and effectiveness.

**Supplementary Information:**

The online version contains supplementary material available at 10.1007/s40841-025-00390-6.

## Introduction

The wellbeing of Aotearoa New Zealand (NZ) adolescents (rangatahi) is a critical concern, with challenges during this period impacting into adulthood (Department of the Prime Minister & Cabinet, 2019; Soutter et al., 2012). Adolescence is a crucial developmental stage with physical, emotional, and social changes, making it a time of vulnerability and growth (Blakemore & Robbins, 2012; Crone & Dahl, 2012; Petersen, 1988). In the context of secondary education, the wellbeing of adolescents plays a pivotal role in shaping their academic success, social interactions, health and happiness, and overall quality of life (Konu & Rimpelä, 2002; Waters, 2015; Moore et al., 2017; Steptoe, 2019). In NZ, many adolescents are experiencing a poor quality of life (Department of the Prime Minister and Cabinet, 2019; Soutter et al., 2012). The Youth19 report highlights declining adolescent mental health compared to previous surveys and that there is “persistent and growing mental health inequity between Māori and other ethnic groups”, with rangatahi Māori having higher rates of depressive symptoms and suicide attempts compared to other ethnic groups NZ (Fleming et al., 2020, p. 1).

Secondary schools are ideally positioned to support rangatahi wellbeing, but time constraints and adolescent development complexities create challenges for educators (Hargreaves, 2013; Özdemir et al., 2016). Tāhūrangi, the NZ Curriculum addresses mental health through the Health and Physical Education learning area, with hauora as a core concept (Ministry of Education [MoE], 2007). Hauora is a Māori philosophy of holistic health and wellbeing that is represented by the model Te Whare Tapa Whā, which recognises the interconnectedness of the physical (Taha tinana), mental and emotional (taha hinengaro), social (taha whānau), and spiritual (taha wairua) dimensions and connection to the land (whenua) (Durie, 1998). The curriculum promotes key competencies, such as relating to others and managing self, which are essential for positive mental health (MoE, 2007). However, assessing these competencies remains largely informal, with less emphasis as learners progress through secondary education. Effective and time-efficient tools for understanding and promoting rangatahi wellbeing will help facilitate the integration of hauora into the classroom.

The Meke Meter™, an Indigenous, image-based self-reflection tool (Forrest et al., 2019), offers a unique approach to assessing adolescent hauora. Existing wellbeing tools did not accommodate people with low literacy or adequately capture cultural and spiritual dimensions. Developed by Māori for Māori, the Meke Meter™ addressed the need for a culturally relevant, holistic, and accessible tool for measuring wellbeing; prompting users to score themselves on different aspects contributing to their overall health and wellbeing, enabling them to identify areas for improvement, set goals, and track progress over time (Forrest et al., 2016, 2019). This self-directed approach fosters a sense of agency and autonomy in wellbeing-related decisions. The Meke Meter™ has demonstrated utility in tertiary education, health, and fitness (Forrest et al., 2016, 2019; Harvey et al., 2019), but its application in secondary schools is unexplored.

This study investigates the suitability and efficacy of the Meke Meter™ in capturing the self-evaluated wellbeing of rangatahi in NZ secondary schools. The integration of tools like the Meke Meter™ can create a supportive environment for the holistic development of students and provide educators with insights into adolescents’ needs and challenges (Education Review Office, 2016; MoE, 2017). This deeper understanding facilitates targeted interventions, support services, and educational approaches that cater to the diverse needs of students.

This qualitative study involved two distinct secondary education case studies exploring the experiences of rangatahi and teachers with either the paper-based or online version of the Meke Meter™. This study aimed to determine the tool’s user-friendliness, perceived value in promoting self-awareness and goal-setting, and potential for integration into the secondary school curriculum and pastoral care systems. The case studies addressed the following questions (Armstrong, 2022a, 2022b):

1. Is the online or paper version of the Meke Meter™ a suitable and user-friendly interface for rangatahi to self-evaluate their wellbeing?
2. Do classroom teachers feel that the Meke Meter™ can be effectively applied to the wellbeing curriculum?

## Methods

This study employed an exploratory multiple case study design (Yin, 2009) grounded in an interpretative paradigm to centre the lived experiences (Fossey et al., 2002) of rangatahi and teachers regarding using the Meke Meter™. The research was informed by Kaupapa Māori principles, prioritising the Indigenous voice in every aspect of the research, centring Māori perspectives and experiences (Pihama, 2010; Smith, 1999); recognising the significance of pūrākau (personal stories) as a source of knowledge in Indigenous cultures (Ware et al., 2018). The two schools were chosen purposefully due to their high Māori demographic.

**Fig. 1:**
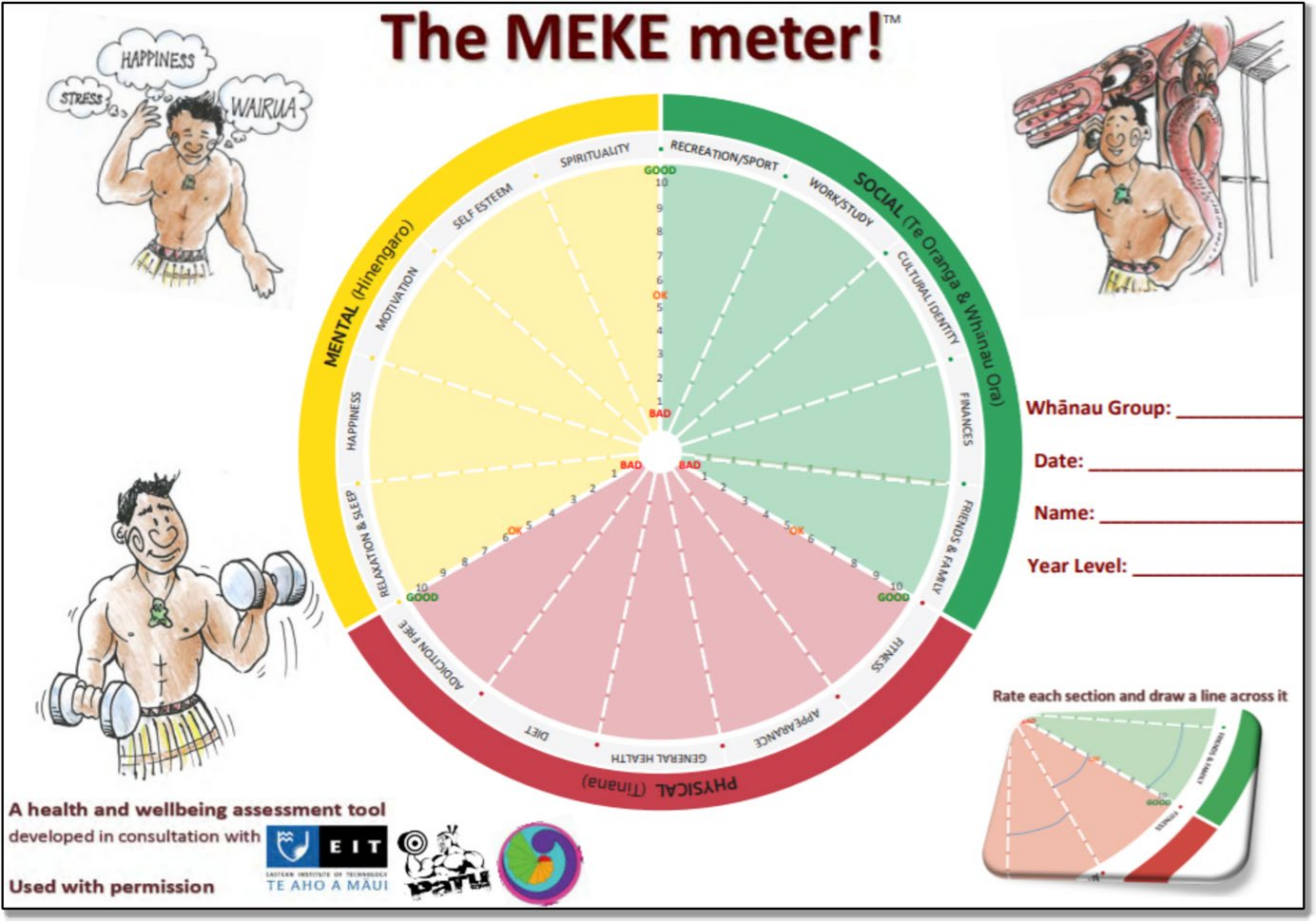
The paper-based Meke Meter™. Adapted for use in a mainstream co-education secondary school’s Whānau groups

*Research Setting and Participants:* The study involved two distinct secondary education case studies within Hawke’s Bay, NZ:

- *Mainstream Co-educational Secondary School* The paper-based Meke Meter™ (Fig. 1) was implemented school-wide by 11 teachers in 24 Whānau groups (pastoral care groups). These groups met daily for 15 min, each with about 12 students from different year levels who remain in the same group throughout their time at school. Participants included 85 students (aged 12–18) who completed the Meke Meter™ weekly for four weeks.
- *Hawke’s Bay Alternative Education Programme (HBAEP)* The online Meke Meter™ (Supplementary Fig. 1) was integrated into the teaching practice of the programme coordinator at an alternative education establishment during regular weekly sessions in a computer classroom. Four out of five enrolled students participated. The HBAEP works with the region’s most disadvantaged rangatahi, many of whom have not attended a mainstream educational setting for over 12 months.

*Data Collection:* This occurred in 2021 and utilised Google Form questionnaires. The student survey consisted of the questions that had previously been used to gain feedback from students in the Patu™ Meke Meter -Use in the Classroom study (Forrest et al., 2019; Table 1). The online format ensured anonymity and encouraged candid feedback, as there was no fear of handwriting being recognised. Feedback was collected from the teachers using questions shown in Table 2. Feedback was obtained from 10 of the 11 Whānau group teachers and one teacher of the HBAEP class.

**Table 1:** Student questions for the paper-based version of the Meke Meter™

Number	Main question	Prompt questions
1	How did you find the Meke Meter™ to use?	How did you find it to understand? How did you find it to fill out?
2	What do you think about the presentation of the Meke Meter™?	What did you think about the colours, layout, use of words and language, pictures etc.?
3	How well do you think the Meke Meter™ reflected your wellbeing?	How well do you think the Meke Meter™ showed how you were feeling about life?
4	Did you find using the Meke Meter™ useful? – If so, how? – If not, why not?	What did you get out of it by filling in the Meke Meter™? Would you do it if it was not part of the class activities?
5	How could the Meke Meter™ be improved?	If you were re-designing a Meke Meter™ would there be any sections you would put in or take out? Would you make it look different or change the way it is filled out?

**Table 2:** Questions for participating teachers about the use of the Meke Meter™ in their classroom practice

Number	Question
1	How did you find administering the Meke Meter™?
2	How did it fit in with the rest of the curriculum?
3	How much explanation did the Meke Meter™ require for the students to be able to fill it out?
4	While your students were completing the Meke Meter™, do you think they understood what each section meant?
5	What questions did your students have while they were filling it out?
6	How could the Meke Meter™ be improved?

*Data Analysis:* Two forms of analysis were employed:

- *Inductive Thematic Analysis* Following Thomas’ (2006) general inductive approach, the questionnaire data were systematically reviewed to identify emergent themes and categories.
- *Critical Analysis using Appreciative Inquiry* This approach aligned with Kaupapa Māori principles and sought to identify positive aspects of the Meke Meter™ and areas for refinement to enhance its effectiveness in promoting wellbeing. Kaupapa Māori principles that align with appreciative inquiry include its strength-based, collaborative approach concerned with structural change and finding solutions to facilitate better pathways and outcomes for whānau, engaging them to drive the research and decide how it might best serve their aspirations rather than focusing on deficits (Coghlan et al., 2003; Cram, 2010).

### Ethical Considerations

Ethical approval was obtained for the project from the EIT Research and Ethics Approvals Committee (REAC 20/12). The school principal approved the incorporation of the Meke Meter™ into standard classroom practice in the mainstream secondary school setting. The Lead Facilitator and Founder of the HBAEP approved the use of the Meke Meter™ for their rangatahi. For both settings, a study description was also included in a school newsletter to the parents, advising them that students would be asked for their voluntary participation but that they could request that their child not be involved. Students received participant information about the study, and a presentation was provided for the teachers about the study, how to use the Meke Meter™, and inviting them to participate. The questionnaires were fronted with the following statement: “This survey is anonymous. No one, including the researcher, will be able to associate your response with your identity. Please do not indicate your name, institution or geographic region. Your participation is voluntary, and you may choose to stop responding at any time during the survey. The completion of the survey indicates your voluntary agreement to participate in this research project.” Copies of the information are available in the appendices of Armstrong (2022a).

## Findings—Thematic Analyses

*Students’ Experiences of the Paper-based Meke Meter™* A total of 85 rangatahi used the paper-based Meke Meter™ tool; 14 filled in the questionnaire. Not all the rangatahi gave feedback on all the questions asked. The responses were read several times and a coding frame developed, and themes and categories identified (Supplementary Table 1): Appeal (themes: Ease of use, Aesthetics), Self-Reflection (themes: Self-awareness, Tracking progress, Goal setting), and Development (themes: Potential improvements, Needed teacher input). For example, the consensus was captured by one participant that the Meke Meter™ was “quite easy to fill out”, “creative”, “good for reflecting” and “useful as it showed what categories I needed to work on the most, and what were good” (R4).

Within the category of Appeal, in general, the students found the paper-based Meke Meter™ aesthetically pleasing with comments such as “Good pictures, good colours” (R4) and “I liked it” (R8) being typical. Comments regarding the Ease of Use of the Meke Meter™ ranged from “it was pretty self-explanatory and simple to understand” (R10) and “fairly easy to use” (R13, R14) to “confusing at the start” (R7) and “once I knew how to do it, it was simple” (R5). Many of the comments could be summed up by one participant who said, “it was confusing at the start, but the teacher explained, and I understood pretty well” (R8).

Within the Self-reflection category, the feedback about using the Meke Meter™ as a self-reflection tool was positive. Self-awareness emerged as a theme, as participants commented about the fact that they can “see how they feel” (R8) and “It was useful, I like to check in with myself to see how I’m feeling” (R14). Some comments highlighted the potential for Goal setting, for example, “it showed me areas that I needed to fix and get better at” (R10). The final theme to emerge within Self-reflection was Tracking progress, it was felt that using the Meke Meter™ was “… like having a little reflection on how your week has been and how connected you are to your family and to your culture” (R6) and that “it would be useful if you wanted to know where you’re at and see where you could improve on” (R2). The cultural relevance of the Meke Meter™ was captured by one student who said it helped them to reflect on “… how connected I am to my family and my culture” (R6).

*Students’ Experiences of the Online Meke Meter™:* Four (n = 4) rangatahi provided feedback. The thematic analysis revealed three main categories: Appeal, Self-reflection, and Development (Supplementary Table 2). The themes contributing to these categories were slightly different from those that emerged from the mainstream setting, with the previously identified ‘Goal-setting’ and ‘Needed teacher input’ themes not emerging and ‘Administration’ newly emerging as a theme contributing to the category of Development.

The consensus from the feedback provided was constructive and could be captured by the comments from one participant, which included “… straightforward”, “simple”, and “… gives us an excuse to stop for 5 min and focus on ourselves and check in” (RO3). The comments regarding the presentation of the online platform were all positive, “looks good” (RO1) and two comments were given about liking the graph after the user had completed their entry “I liked how I got a graph at the end” (RO4) and “the circle was pretty cool” (RO3) and one also stated that they liked “how you can easily compare the different sections of your wellbeing” (RO3). Suggestions for improvement included “it would be good to have suggestions on where I could go if I wanted to improve an area” (RO1) and “gamification” (RO3, RO4).

*Teachers’ Perspectives About the Meke Meter™* The feedback provided by teachers was mostly positive. Themes that formed three categories were identified (Supplementary Table 3), namely: Appeal (themes: Ease of use, Minimal instruction required, Presentation, Reflection); Fit (themes: pastoral, Kāhui Ako focus, curriculum); and Development (themes: Developing educator resources, Developing student resources). Eight of the 10 teachers who provided feedback commented that the Meke Meter™ was easy to use. Comments that supported this observation included “The Meke Meter was quite straight forward once the students knew how to complete it” (K1), “Quite straight forward, once instructions were given, it was easy to use” (K2), “Easy to use and straight forward” (K5) and “pretty easy to administer … once they got it” (K2), “…not a lot of explanation was required” (K9), and “Minimal explanation was required” (K10). Most teachers provided instructions when introducing the Meke Meter™, and there was no need to provide further directions for the subsequent distributions. Whānau Groups consist of rangatahi from multiple year levels, and two teachers’ responses reflected the user-friendly nature of the Meke Meter™ being supportive of different literacy levels as “[the Meke Meter™] was simple and straight forward for any level of student” (K5) and “Even the more literacy challenged students got it once they had the dimensions explained to them” (K8).

Comments about the presentation of the paper version of the Meke Meter™ were overall positive. The colours, ease of understanding and data collection were pleasing characteristics. There were constructive comments regarding the presentation of the Meke Meter™ that could assist in improving the tool. Including “The Meke Meter™ itself is too small relative to everything else on the page” (K3), “the graphics on the bottom right logo etc. should be bottom right with the Meke Meter™ rate on the left as you read left to right and this information is important to complete the circle graph” (K4) and “… if you took out one of the pictures and put a simple explanation of spirituality (and perhaps cultural identity?) It is always one they struggle with” (K8).

The Meke Meter™ appeared to allow teachers to gain insight into the lived experiences of rangatahi, which could enable them to alter their teaching pedagogy to better cater pastorally to their students’ needs. Comments such as “I realised some things about my students that I needed to address like [student name] and [gender] very low self-esteem. I kind of knew it in the back of my mind but this was evidence from [gender] that [gender] could be better and brought it to the fore” (K8) and “… I knew some of my students were troubled, but it gave me a measure of how troubled they were relative to others of their age or gender” (K3). Comments that aligned to tracking progress over time included “if it were to be used consistently, you could see if there are patterns in days, weeks, months … you can also feel good about those things that are good” (K2) and “interesting to see how students rated themselves and the variation between weeks” (K5).

One of the goals of the Kāhui Ako was to focus on student wellbeing and three teachers commented that the Meke Meter™ “fits in with our focus on wellbeing” (K1); “school wellbeing focus” (K3) and “links with our wellbeing focus” (K8). Links were made to the curriculum also, which were reflected in the comments that the Meke Meter™ was “Particularly appropriate for Health” (K5) and “We could relate it back to Te Whare Tapa Whā” (K1), and three teachers highlighted how the Meke Meter™ could support all curriculum areas, saying “Wellbeing is in all parts of the curriculum” (K2), “…can relate to all areas” (K7) and “…it fits well as it over arches everything” (K6).

Some teachers’ comments suggested that the development of student and teacher resources would support the use of the Meke Meter™ in the classroom. Comments that aligned with this included “Some needed clarification on ‘spirituality’, ‘finances’, ‘addiction’” (K6) and that more “in-depth pre-learning would be beneficial” (K8).

## Appreciative Inquiry – A Critical Analysis and Discussion

**Fig. 2:**
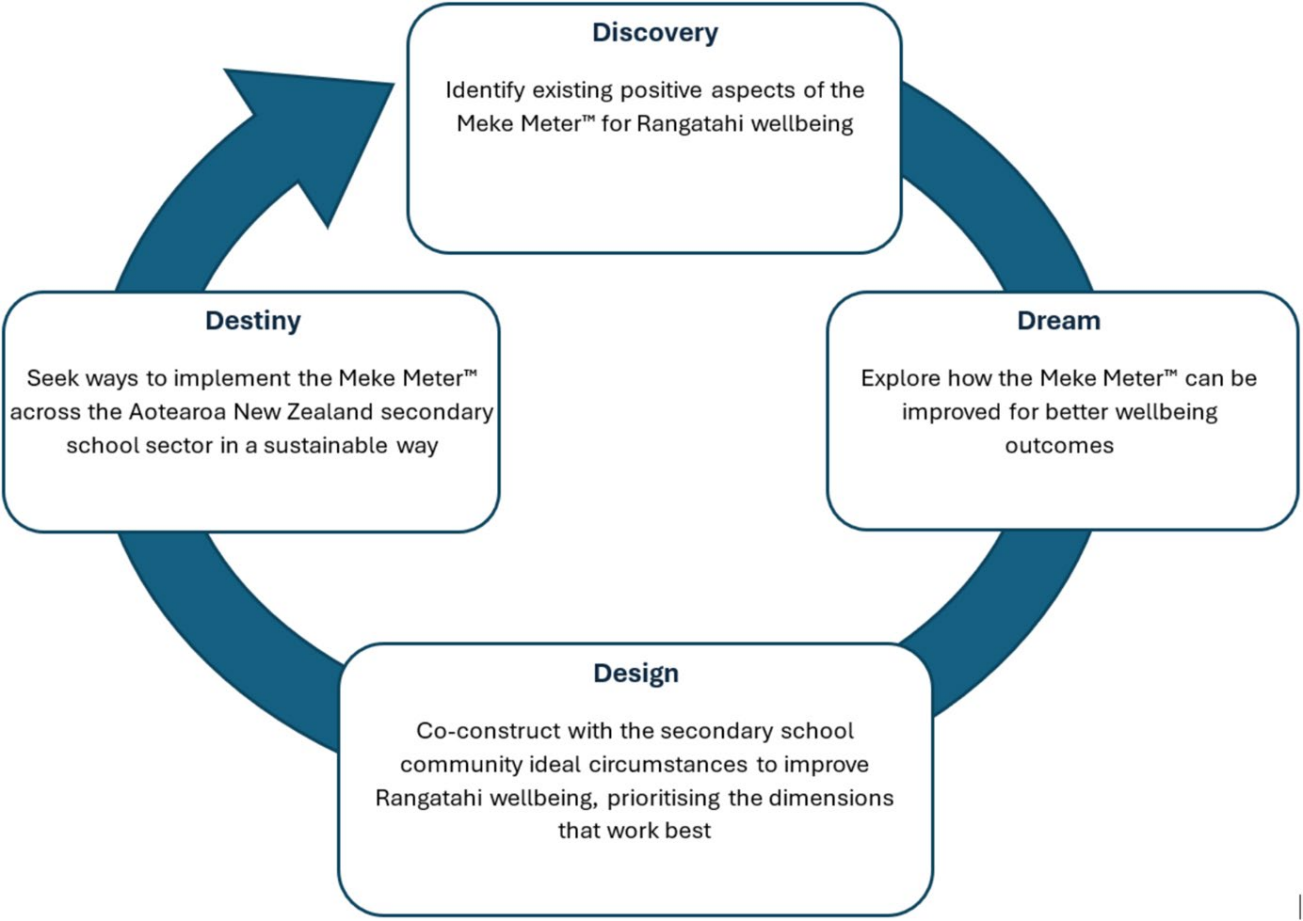
An appreciative inquiry relational 4-D cycle

In the second form of analysis, the data were examined using appreciative inquiry, a strengths-based approach aligned with Kaupapa Māori principles (Cram, 2010). This method, which uses four phases (Discovery, Dream, Design, and Destiny) emphasises solutions and positive change and allows the successes of the Meke Meter™ to be explored alongside other evidence (Fig. 2). This approach blurs the lines between findings and discussion, which are reported in separate sections by convention. However, in doing this, the appreciative inquiry approach acknowledges the interconnectedness of all things present, past, and future. This aligns with Te Ao Māori and Mātauranga Māori, emphasising the interconnectedness of all things, including people, nature, and the spiritual realm.

### Discovery Phase

Mā te kimi ka kite, Mā te kite ka mōhio, Mā te mōhio ka mārama

Seek and discover, discover and know, know and become enlightened

Thematic analysis highlighted the Meke Meter™’s potential in secondary education. The tool’s quick and easy administration enabled successful integration into teaching and provided regular wellbeing snapshots, aligning with evidence suggesting the synergy between health, wellbeing, and education (Bradley & Greene, 2013; Littlecott et al., 2018a, 2018b). Unlike traditional methods, where NZ schools rely on once-off large-scale school-nurse-led psychosocial screening surveys like HEEADSSS and teacher observations to manage student wellbeing to inform policymaking, the Meke Meter™ enables ongoing monitoring, capturing wellbeing fluctuations (Thabrew et al., 2019). Facilitating student voice promotes co-construction of learning and improved outcomes (Cook-Sather, 2020; Robertson, 2017). This necessitates a power-sharing shift, recognising students’ central perspectives (Cook-Sather, 2006; Mayes et al., 2017). As Berryman, Eley, and Copeland (2017) explain that “By altering the power relationships and pedagogies within classrooms, teachers found that the students’ cultural values could enhance cognitive engagement and subsequent achievement” (p. 483).

The Meke Meter™ also facilitates pastoral support, appealing to teachers and crucial since poor wellbeing can hinder learning (Bascia, & Hargreaves, 2014; Hargreaves, 1994). Student wellbeing can change radically from day-to-day and week-to-week on minor and major issues, which is not captured by once-off psychosocial screening. However, tracking wellbeing in secondary schools where students have multiple teachers with limited time to support student wellbeing (Bascia, & Hargreaves, 2014; Hargreaves, 1994). Using the Meke Meter™ supports the understanding that student–teacher relationships and peer relationships are predictive of wellbeing (Littlecott et al., 2018a, 2018b; Moore et al., 2017). Furthermore, the health behaviours of adolescents such as physical activity, diet, and substance use, as well as other outcomes such as mental health and subjective wellbeing are patterned by social and economic characteristics that track throughout the life course (Boreham, et al, 2004; Moore & Littlecott, 2015; Moore et al., 2015; Park, 2004). Thus, the Meke Meter™ aids in identifying support needs, fulfilling an ethical teacher responsibility.

Embedding the frequent collection of students’ voices into teaching practice involves training and positioning students to be able to “identify and analyse issues related to their schools and their learning that they see significant” (Fielding & Bragg, 2003, p.4). Thus, empowering students to “speak and act alongside credentialed educators as critics and creators of educational practice” (Cook-Sather, 2018, p. 17). Regular use of the Meke Meter™ fostered self-awareness and reflection, crucial for goal setting (Travers et al., 2015). In addition, Travers et al. (2015) noted the importance of identifying psychological factors such as a lack of self-confidence or low self-esteem that may undermine a student’s achievement of their goals. The Meke Meter™ enables identifying growth areas and potential barriers, integrating an inquiry-based process for wellbeing enhancement (Copland, 2003).

Te Kete Ipurangi encourages reflective practice, stating that “when students become reflective about the teaching and learning process, they are strengthening their own capacity to learn” (MoE, n.d. para. 1). Self-reflection improves critical thinking and self-knowledge (Rusche & Jason, 2011), and aids in goal setting for personal growth, impacting self-esteem and self-efficacy (Rickard et al., 2016; Travers et al., 2015). This contributes to learner autonomy and emotion regulation, crucial in adolescence for facilitating self-directed learning and self-actualisation (Gross & Thompson, 2007; Lennarz et al., 2019; Naude et al., 2014). In this study, teachers noted that the Meke Meter™ facilitated student self-assessment and reflection on various life aspects impacting their overall school experience. This enabled students to identify areas needing more or less focus.

Emotion regulation promotes psychological flexibility, resilience, and wellbeing and is central to psychosocial functioning and mental health (Gross & Thompson, 2007; Lennarz et al., 2019). These aspects impact the learning process (Ingleton, 1999). Emotion shapes and is shaped by social relations, self-esteem, and identity and plays a complex and dynamic role in shaping the learning experience. Using a tool such as the Meke Meter™ to guide and inform teaching and learning could assist students in their emotion regulation by offering an opportunity to recognise strengths and set goals for improvement.

Overall the Discovery phase about the Meke Meter™ supports the recognised link between wellbeing and learning. The Meke Meter™ enables students to identify how the various aspects of their lives impacted on their overall health and wellbeing and regular use within the secondary school context enables user-friendly wellbeing monitoring in a culturally considered manner, evidenced by both student and educator perspectives. The cultural relevance of the Meke Meter™ was not specifically examined in this research and is a limitation of the present study.

### Dream Phase

The Dream phase of appreciative inquiry involves “the creation of a vision that brings to light the collective aspirations of stakeholders” that emerged in the Discovery stage (Sullivan, 2004, p. 224). Thematic analyses of feedback from rangatahi and teachers show the Meke Meter™’s potential to aid secondary school students navigate adolescence. The positive response has led to a dream of further developing the Meke Meter™ into a tool that transcends its original purpose. Considering diverse stakeholder perspectives, this dream envisions the Meke Meter™ as a catalyst for building capacity, improving wellbeing resources, and facilitating widespread implementation in all NZ secondary schools.

Nationally, the Meke Meter™ can support the six outcomes in the NZ Government’s Child and Youth Wellbeing Strategy which envisions NZ as the best place in the world for children and young people, with education playing a crucial role (Department of the Prime Minister and Cabinet, 2019; MoE, 2021). The Strategy emphasises young people’s desire to be loved, safe, and nurtured; to have basic needs met; to be happy and healthy; to learn and develop; to be accepted, respected, and connected; and to be involved and empowered. These outcomes, reflecting young people’s priorities, encompass necessary factors for their wellbeing. Currently, no system is in place to measure these outcomes, and the Meke Meter™ could potentially fill this gap.

In addition to the national strategy, the MoE’s National Education and Learning Priorities (NELP) acknowledges the responsibility of schools to prioritise learners and their families. This involves ensuring that learning environments are safe, inclusive, and free from discrimination and that schools maintain high aspirations for all students, partnering with families and communities to provide culturally responsive education (MoE, 2020). Education is a key construct for promoting wellbeing effectively and sustainably, equipping students to shape positive change (Sterling, 2001). Therefore, promoting student wellbeing in schools goes beyond improving academic achievement or reducing the burden of ill health. It’s about empowering students to lead fulfilling and flourishing lives, and schools are crucial settings for fostering this wellbeing. The Meke Meter™ implemented school-wide, can make student wellbeing integral to the school’s curriculum, culture, structure, relationships, and services. It can also amplify student perspectives, shifting away from the traditional reliance on adult viewpoints (Mitra, 2018) and emphasising student competence, abilities, and aspirations.

From the perspective of NZ secondary school educators, the Meke Meter™ aligns with the Teaching Council of Aotearoa NZ Our Code, Our Standards (2017). The thematic analysis revealed that the tool has the potential to monitor adolescent wellbeing, enabling teachers to “… work in the best interests of learners by promoting the wellbeing of learners and protecting them from harm” (Education Council, 2017, p. 10). Teachers found the tool useful for pastoral care, providing insights into individual and group wellbeing and serving as a conversation starter. Its alignment with the curriculum’s emphasis on Te Whare Tapa Whā was also noted. It also supports non-written reflection, addressing literacy disparities (Forrest et al., 2019). By gaining insights into how students rate different aspects of their lives, teachers are better equipped to provide guidance, support, and make connections between their students’ lives and their learning.

From the student’s perspective, the Meke Meter™ can facilitate self-reflection and goal-setting aligned with their aspirations, thus aiding in developing a positive identity. It helps students recognise imbalances in their wellbeing, a skill that requires practice and is crucial for navigating adolescence. While NZ secondary schools prioritise literacy, numeracy, science, technology, and physical activity as essential learning areas (MoE, 2015), socio-emotional skills are also included in the curriculum as "key competencies" which aim to prepare young people for lifelong learning and engagement in society, however, their assessment remains informal (Gillespie et al., 2013; MoE, 2015). Although exemplars and discussions highlight the importance of integrating key competencies into academic learning (Davis et al., 2013; Gillespie et al., 2013; Hipkins et al., 2014; MoE, 2018), the socio-emotional component of the key competencies remains subsumed in classroom-based teaching. Contemporary educational policies still measure short- and long-term success completely in terms of academic performance, and shifting the measurement of learning success to encompass wellbeing has positive implications for rangatahi in terms of viewing learning more holistically and acknowledging the importance of identity development. Integrating the Meke Meter™ into the national curriculum could shift the focus onto these vital competencies and contribute to a more holistic assessment of student success. This notion is consistent with the findings of Forrest et al (2019) who stated that “the use of the Meke Meter in classrooms was able to record positive changes in the health of students creating a measure of success beyond that of traditional mainstream measures” (p.16).

The Dream section presents evidence that using the Meke Meter™ in secondary schools can promote student wellbeing and fulfil key stakeholders’ aspirations, namely the NZ Government, MoE, NZ secondary school educators and NZ secondary school students. However, it is important to acknowledge that this study did not explore the perspectives of parents or the wider community, highlighting an area for future research.

### Design Phase

This study employed two versions of the Meke Meter™ (the paper-based and online versions) in two different settings with two different population sizes. It revealed common feedback, including the desire for goal-setting features. The online users also noted that gamification would make the Meke Meter™ more engaging. These findings echo those of Armstrong (2022b), whose research also suggested the value of goal-setting, gamification, and regular notifications in enhancing the usability of the online Meke Meter™. Educators also needed clearer explanations of subsections and additional supporting resources. While the paper-based version was appreciated for its aesthetics (which differs to the online platform), it presented challenges in data tracking and analysis. The online platform, however, offers development opportunities that the paper version cannot, such as audio or pop-out text explanations and the potential for goal-setting, notifications, and gamification features.

Given the ubiquity of mobile devices and their potential for personalised health tracking, future development should prioritise a mobile app (Consolvo et al., 2014), that is rangatahi-specific, as opposed to the further development of the current web-based application. This app could seamlessly integrate wellbeing monitoring into adolescents’ lives, addressing barriers such as time constraints and motivation (Dute et al., 2016; Madden et al., 2013). Furthermore, embedding such interventions within existing structures like schools has proven to be more effective (Kohl et al., 2013). National administration of the app, potentially by the MoE, would facilitate collecting and analysing valuable data to inform wellbeing policies and interventions. However, this raises important ethical considerations regarding data privacy and the potential for self-reported low scores to trigger unwarranted interventions.

Teachers are legally bound to provide a duty of care to their students and are required to consider sharing information if it may help protect a student from harm. In secondary schools, students frequently move in and out of vulnerability; therefore, tracking changes can be useful in detecting and managing triggers. However, this has implications when administering the Meke Meter™ in the classroom when a child scores themselves low in a category and for deciding when reporting is appropriate. Evidence’s credibility can be challenged if revealed through a self-reflection wellbeing instrument. For example, two students may score themselves the same low grade in self-image. However, one is pessimistically minded, and the other is at risk of self-harm. Thus, this highlights important areas for further research 1) the need to couple the Meke Meter™ data from an individual with information about their personality type and 2) the creation of appropriate teaching resources and support material to assist teachers in administering the Meke Meter™ into their classroom practise and in providing the appropriate support to students. There is also scope for research to develop or connect students to resources to facilitate autonomy and empower them to meet any goals they set for themselves in response to using the Meke Meter™.

The Design phase will necessitate comprehensive consultation with various stakeholders, including rangatahi, educators, whānau, the wider community, the MoE, health professionals, and Iwi. This collaborative approach will ensure that the Meke Meter™ evolves into a holistic, well-resourced, and culturally safe tool that helps to equip rangatahi to take ownership of their wellbeing.

### Destiny Phase

The Education Review Office *Wellbeing for Success* resource emphasises schools’ role and responsibilities in promoting student wellbeing (Education Review Office, 2016). While learning, wellbeing, and socio-emotional development have been extensively researched internationally (for examples see Biggeri & Santi, 2012; Cahill & Dadvand, 2020; Collie et al., 2017), the socio-emotional component of the NZ Curriculum’s key competencies remains under-explored in NZ educational literature. Though these competencies are foundational to the curriculum’s vision of fostering confident, connected, lifelong learners (MoE, 2007) they often remain implicit aspirations rather than explicit components of holistic education.

Further development of the Meke Meter™ into a rangatahi-specific mobile app, with an educator-accessible backend and integration within the NZ curriculum, could bring these key competencies to the forefront. By enabling students to measure, reflect on, and track their progress, the Meke Meter™ would align with the NZ Government’s Child and Youth Wellbeing Strategy and promote a proactive approach to wellbeing (Department of the Prime Minister and Cabinet, 2019). Providing access to the Meke Meter™ and wrap-around resources for teachers and students would begin to address some of the growing mental health concerns with regards to NZ rangatahi and help to equip our young people to develop resilience and coping strategies, which in turn will alleviate pressure on mental health services. This will shift away from the ‘ambulance at the bottom of the hill’ scenario and place wellbeing education ‘at the top of the hill’ as the primary prevention. To achieve this investment will be required into further research and development of the rangatahi-specific Meke Meter™ app along with partnerships being established with MoE to roll out its use to secondary schools by providing professional development for teachers and partnership with other Government Departments such as the Ministry of Health and Ministry of Social Development to facilitate the development of appropriate resources and monitor the back end data about rangatahi wellbeing to inform Government policy.

## Summary and Conclusion

In concluding, the importance of self-reflection to te ao Māori is captured in the following whakataukī,

Titiro Whakamuri, Kōkiri Whakamua

Look back and reflect so you can move forward.

Self-reflection is crucial for personal growth and wellbeing, and this research reinforces the significance of holistic learning in schools, advocating for a shift beyond academic achievement to prioritise student wellbeing. Recognising imbalances in wellbeing is essential for early intervention and preventing negative escalation. Despite their potential for nurturing wellbeing, schools often contribute to student stress through academic pressures and complex social dynamics. This study highlights the need for a culturally responsive approach to address the unique wellbeing needs of NZ rangatahi. Although the sample numbers are small, this research marks a significant step towards successfully implementing a culturally responsive, adolescent-specific wellbeing tool within the NZ education system. The Meke Meter™ is a promising tool for promoting self-reflection and equipping students to take ownership of their wellbeing. This research explored its applications in secondary education, gathering valuable feedback from both teachers and students. We conclude that implementing the Meke Meter™ in secondary education settings represents an innovative approach to supporting and enhancing adolescent wellbeing within the secondary school context. By providing students with a structured framework for evaluating their wellbeing and facilitating open dialogues around mental health and self-care, the Meke Meter™ serves as a catalyst for promoting positive attitudes towards health and wellbeing among rangatahi. Future development should involve co-design with stakeholders, ensuring the Meke Meter™ evolves into a comprehensive system that supports rangatahi in thriving and flourishing to live their best possible lives.

## Supplementary Information

Below is the link to the electronic supplementary material.

Supplementary file1 (DOCX 351 KB)

## Data Availability

Data is available on request.
