# Supplementary material for: Enhancing Rangatahi Wellbeing in Secondary Education Through Implementation of the Meke Meter™
Source: N Z J Educ Stud. 2025 Jun 2;60(2):377–96. doi: 10.1007/s40841-025-00390-6 (PMC12657541; doi:10.1007/s40841-025-00390-6)
Supplement: Supplementary file 1 — Supplementary file1 (DOCX 351 KB) [file 40841_2025_390_MOESM1_ESM.docx]

Supplementary Fig. 1 The online Meke Meter™. The sub-sections are rated using a sliding scale, as shown in a) and b), which shows how the results are displayed.

1.
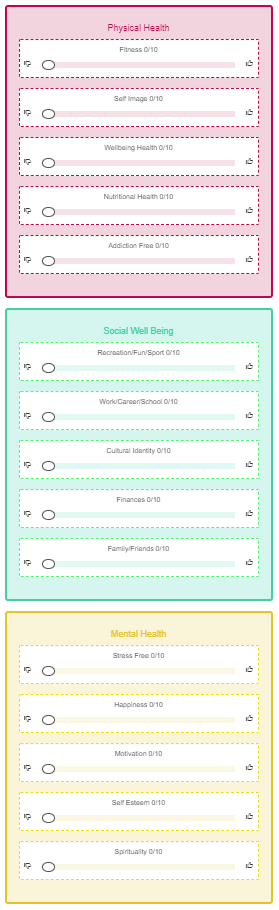
 b)
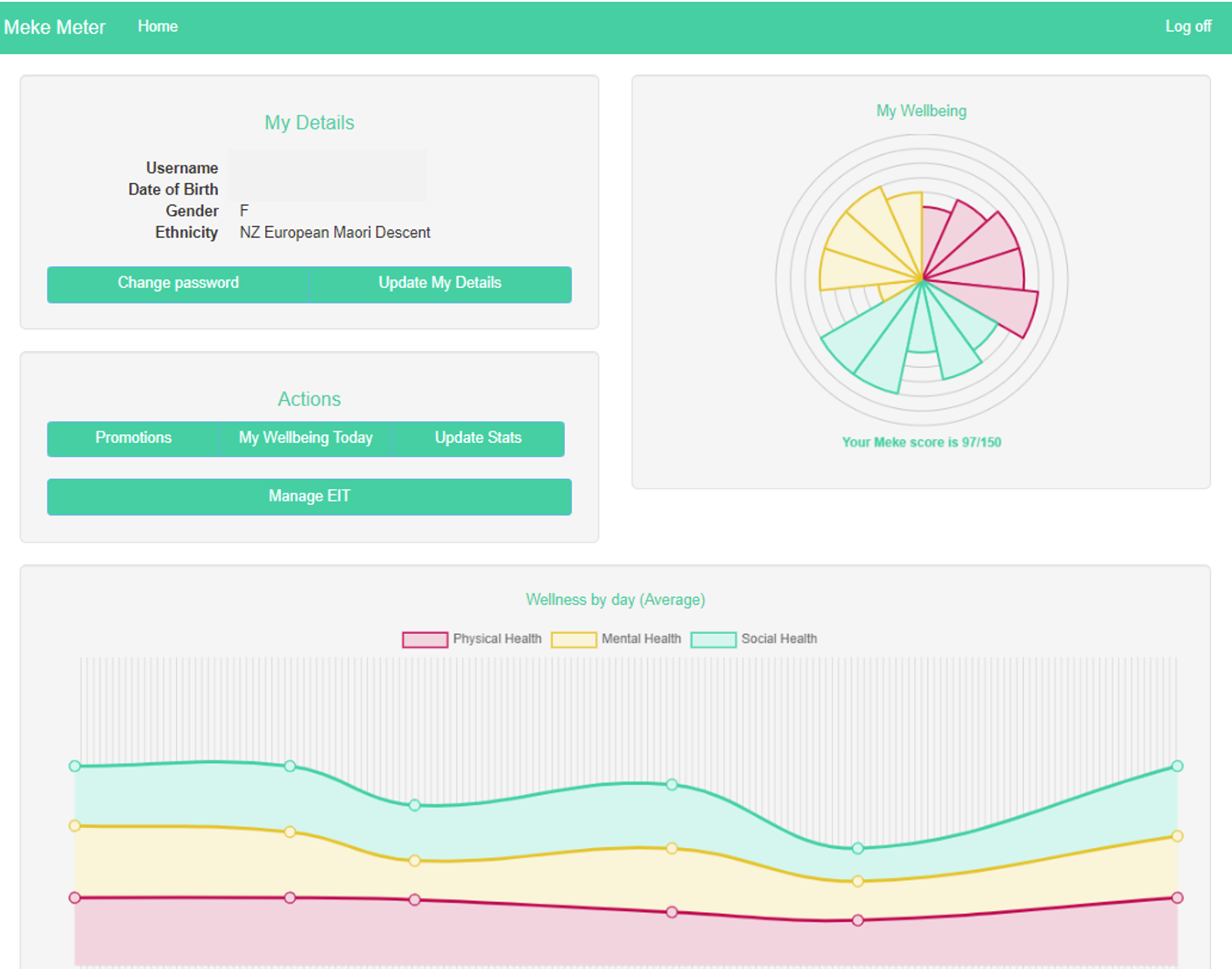


Supplementary Table 1 Inductive thematic analysis of student feedback regarding the use of the Meke Meter™ in a Whānau Group environment (n = 14)

| Category | Theme | Quotes |
| --- | --- | --- |
| Appeal | Ease of use | “It was pretty easy because it's pretty simple” R7  “I found it pretty easy to use and to understand” R9; “The Meke Meter was easy to use and to understand” R12  “Fairly easy to use” R13; “easy to use” R14  “Yes, once I knew how to do it, it was simple” R5  “Easy when my teacher explained it” R8  “I understood it well, the patai were explained well enough for me to answer … I knew what I needed to do” R10  “It was pretty self-explanatory and simple to understand, I didn't find it confusing” R12  “Very easy to understand, the Meke Meter wasn't complicated, and the instructions given were clear” R13  “It was easy to understand and pretty straight forward … didn’t take much time at all” R14  “Easy R2, R5, R8 … to fill out, no complaints about the format of the Meke Meter” R13  “Quite easy to fill out R4, R6 … because of how it was laid out” R12 |
|  | Aesthetics | “Creative” R1, R3  “Good” R2, R7, R9  “Creative, colourful” R3, R4 “… pictures are good because they explain some of the categories” R4  “Amazing” R5, R6  “Well-presented” R6, R10; “Presentation was good” R13, R14; “… you can understand and see where things are and how it works” R10  “I like it” R8  “Bright and interesting” R3  “Good pictures, good colours” R4  “The colours could probably match each third”. “They're all primary colours, the images work well, there are physical images, mental images, etc”. “The images represent parts of the Meke Meter” R10  “I like the colours, it’s very bright and colourful which makes it cool to look at and easy to focus on” R13  “I liked how there were different colours for each category and how there was an example at the bottom to help explain how we were supposed to use it”. “I liked the images that were used as well” R12  “It's colourful, simple to use once you understand it” R11  “The colours help to keep the sections together” R7 |
| Self-reflection | Self-awareness | “I found it useful to help understand people's mental and physical health and to look at it and hopefully improve” R1  “Useful if you wanted to know where you're at and see where you could improve on” R3  “Useful as it showed what categories I needed to work on the most, and what were good” R4  “It was good for reflecting” R4  “It was very helpful to view my life at the time I was filling it out”. “I didn't generally look at my life from a view point but now I think to myself, "How am I doing?" R5  “… It was like having a little reflection on how your week has been and how connected I am to my family and my culture” R6  “… made me realise that there are things I need to change” R7  “I can see how I feel” R8  “I found it helpful because I could see for myself on paper how I was in certain areas” R12  “It was useful, I like to check in with myself to see how I'm feeling” R14  “I did find the Meke meter useful as it made me think about how I felt and made me more aware of my health” R13 |
|  | Goal setting  Tracking progress | “It would be useful if you wanted to know where you're at and see where you could improve on” R2  “I found that it was useful and that I was able to identify what I needed to work on” R10  “It showed me areas that I needed to fix and get better at” R10  “… like having a little reflection on how your week has been and how connected you are to your family and to your culture” R6  “It lets people track how they are going” R9 |
| Development | Potential improvements | “Be more easier to understand, like using graphs or writing the instructions on the side” R3  “Be clearer about how and where to draw the lines, maybe include what the different sections are and relate to” R4  “Maybe having an area where you can write about your bad feelings if you wanted to” R6  “Giving rewards for using it?” “There was a section on finances, we do not learn about finances in school, it would be good to, what about a section on goal setting?” R14 |
|  | Needed teacher input | “Once I knew how to do it, it was simple” R5  “It was confusing at the start, but the teacher explained and I understood pretty well” R7  “Easy when my teacher explained it” R8  “The patai were explained well enough for me to answer” R10  “A few instructions were needed” R3 |

Supplementary Table 2 Inductive thematic analysis of student feedback regarding the use of the online Meke Meter™ (n = 4)

| **Category** | **Theme** | **Quotes** |
| --- | --- | --- |
| Appeal | Ease of use | “It was easy” RO1 “pretty straightforward”, RO3, “I found it easy to fill out”, RO4  “Good” RO2  “The slider made it easy” RO1, “I liked the slider” RO4  “Simple to fill out” RO3 |
|  | Aesthetics | “Looks good” RO1  “Pretty basic really” RO2  “I liked how I got a graph at the end”, RO4 “… the circle was pretty cool”, RO3 |
| Self-reflection  Development | Tracking progress  Self-awareness  Potential improvement  Administration | “I like how you can easily compare the different sections of your wellbeing” RO3  “… very useful, I liked checking in with myself to see how I was doing, especially when I have a lot of things going on at home” RO1  “… it gives us an excuse to stop for 5 minutes and focus on ourselves and check in” RO3, “I liked having time to check in with myself and really think about how I was feeling in each of the areas”, RO4 and “try to see why I’m feeling certain ways” RO2  “It provides a tool that shows you where you might need to focus for a little bit” RO3  “It gave me the opportunity to reflect on how things were for me without feeling like anyone was going to judge me” RO4  “It would be good to have suggestions on where I could go if I wanted to improve and area” RO1 “… where can we go if we want to improve certain areas”, RO4  “Give us a reason to continue to use it” RO2 “I would like to see some rewards for using it. Like vouchers or discounts or food or something like that”, RO3  “… gamification” RO3, RO4  “… more information outlining the benefits of filling out the Meke Meter to motivate people to use it regularly” RO3  “I would like to see some rewards for using it. Like vouchers, or discounts or food or something like that” RO4  “Have more pictures and different fonts etc” RO1, “Could have pictures and use different font styles”RO2  “Have moving gifs” RO2  “Signing up took a bit of time” RO3; “It took a while to sign up” RO4 |

Supplementary Table 3 Inductive thematic analysis of the Whānau Group teachers regarding the use of the Meke Meter™ in the classroom environment (n = 10)

| **Category** | **Theme** | **Quotes** |
| --- | --- | --- |
| Appeal | Ease of use | “Quite straight forward once the students knew how to complete it” K1, “Quite straight forward, once instructions were given, it was easy to use” K2; “Easy to use and straight forward” K5  “It was pretty easy to administer … once they got it, it was easy after that” K2  “It was easy to use” K3, K7, K8, K9, K10  “It was simple and straight forward for any level of student” K5  “Very good and easy” K7  “Easy to follow and fill out” K7  “Easy to administer” K8 |
|  | Minimal instructions required | “Students only had to be shown once and they were on to it” K1  “Very little explanation needed” K2; “… I explained the first time and they were right” K5  “Not a lot of explanation was required” K9; “Minimal explanation was required” K10  “Even the more literacy challenged students got it once they had the dimensions explained to them” K8  “… in some ways, caters to all communication styles” K10 |
|  | Presentation | “I like the overall presentation” K1  “Good presentation” K2  “Good, colourful and clear … not too busy” K5  “Spot on” K7  “Really good, the students love the colours and the ease of inputting data” K8  “Looks great” K9  “Very clear and easy to understand” K10  “Colours are good” K2 |
|  | Reflection | “Good for students to self-assess and reflect on the different aspects of their lives, which impacts on all of their subjects, extra-curricular activities and interactions with others” K5  “It is useful to have a think about how you are feeling and maybe reflect on why you are feeling that way” K2  “Students can see what areas they need to spend more time on” K7 |
| Fit | Pastoral | “More of a general pastoral tool than an educator tool” K4  “Great fit during whanau group time where checking in on students and their wellbeing is part of the kaupapa” K9  “I realised some things about my students that I needed to address like [student name] and [gender] very low self-esteem. I kind of knew it in the back of my mind, but this was evidence from [gender] that [gender] could be better and brought it to the fore” K8  “It was useful to see how students rated themselves, which allowed for follow up conversations” K1  “If it were to be used consistently, you could see if there are patterns in days, weeks, months … you can also feel good about those things that are good” K2  “… I knew some of my students were troubled, but it gave me a measure of how troubled they were relative to others of their age or gender” K3  “Interesting to see how students rated themselves and the variation between weeks” K5  “Generated the opportunity to have one on one chats” K6 |
|  | Kāhui Ako^*^ focus | “Fits in with our focus on wellbeing” K1; “School wellbeing focus” K3; “Links with our wellbeing focus” K8 |
|  |  |  |
|  | Curriculum | “Particularly appropriate for Health” K5  “Wellbeing is in all parts of the curriculum K2 … can relate to all areas” K7  “Fits in beautifully with Health” K8  “It fits well as it over arches everything” K6  “Aligns with Te Whare Tapa Whā” K8  “We could relate it back to Te Whare Tapa Whā” K1 |
| Development | Developing educator resources | “Spiritual confused with religious beliefs … addiction being limited to drinking, smoking, or drugs etc” K4  “Some needed clarification on 'spirituality', 'finances', 'addiction' etc” K6; “… we had discussions about 'addictions' and 'spirituality' K9  “More in depth pre-learning would've been beneficial” K8 |
|  | Developing student resources | “It really has such huge possibilities for our students. Training up the teachers is key. If wellbeing is going to continue to be a schoolwide focus, then this will be great for schools BUT it has to have a good 'why' and 'how'” K8  “… it would be neat if there was some way for [students] to track their own results in a journal type thing where they can set goals and comment on their enablers and barriers and how they would work to overcome them” K8  “The Meke meter itself is too small relative to everything else on the page” K3  “The graphics on the bottom right logo etc should be bottom right with the Meke Meter rate on the left as you read left to right and this information is important to complete the circle graph” K4  “… if you took out one of the pictures and put a simple explanation of spirituality (and perhaps cultural identity?) It is always the one they struggle with” K8  “… it would have been good for them to have seen their results in a way that perhaps showed the that they were more troubled than others (without me telling them) so that we could discuss this all a bit more without me being the *‘know all’*” *K3* |

^*^Kāhui Ako (Community of Learning) is a group of education and training providers which aim to help students to achieve their full potential (<https://www.education.govt.nz/communities-of-learning/about/> ).
